# Double Trouble: Concomitant Carpal Tunnel Syndrome and Guyon’s Canal Syndrome

**DOI:** 10.7759/cureus.96020

**Published:** 2025-11-03

**Authors:** Josephine Nwankwo, Austin Driskill, John Nguyen, Cami Staker, Omar Selod

**Affiliations:** 1 Medicine, Baylor Scott and White Health, Fort Worth, USA; 2 Physical Medicine and Rehabilitation, Texas College of Osteopathic Medicine, Fort Worth, USA

**Keywords:** carpal tunnel syndome, cervical radiculopathy, dorsal ulnar cutaneous nerve, electrodiagnostic study, guyon's canal syndrome, ulnar nerve entrapment

## Abstract

Carpal tunnel syndrome (CTS) and Guyon’s canal syndrome are distinct mononeuropathies of the median and ulnar nerves, respectively, but their simultaneous occurrence is rare. We present the case of a 54-year-old female who presented with numbness and tingling in both hands. Her physical exam revealed intact sensation bilaterally, full strength, and no muscle atrophy. Electrodiagnostic testing confirmed right-sided CTS and ulnar nerve entrapment at Guyon's canal. Normal latency and amplitude of the dorsal ulnar cutaneous nerve (DUCN) helped localize the lesion distally. The case highlights the importance of comprehensive electrodiagnostic testing, including DUCN evaluation, in identifying concomitant pathologies that may be overlooked clinically. Recognizing these pathologies is important for guiding management, particularly when overlapping symptoms are present.

## Introduction

The median nerve travels through the carpal tunnel to innervate the thumb, index, middle, and radial half of the ring finger. Compression of this nerve can lead to carpal tunnel syndrome (CTS), characterized by pain, numbness, and tingling in these digits. If symptoms are severe or persist, they may progress to motor weakness and muscle atrophy. CTS is the most prevalent focal mononeuropathy and accounts for 90% of all neuropathy cases, with an incidence of under 5% in the general population [1]. This syndrome predominantly affects patients aged 40 to 60 years and is three times more prevalent in females [1]. Other known risk factors of CTS include obesity, diabetes, hypothyroidism, autoimmune diseases, and pregnancy [1]. Symptoms of CTS tend to worsen in the evening and early morning but improve as the day progresses. Repetitive activities, such as typing, can also exacerbate symptoms [1].

The ulnar nerve enters the hand through Guyon’s canal. Compression of the nerve at this location leads to Guyon’s canal syndrome (GCS), which can cause numbness, tingling, and pain in the fourth and fifth fingers and may progress to motor weakness. The incidence of clinical GCS in the general population is extremely rare, with only a few case reports and brief case series [2]. A majority of GCS cases occur acutely in cyclists, leading to the terms "cyclist’s palsy" or "handlebar palsy" [3]. This palsy occurs due to the pressure cyclists place on their wrists and palms from holding the handlebars [3]. Cyclist's palsy, along with ganglion cysts, is the most commonly reported cause of GCS [4].

The incidence of concomitant GCS and CTS has been rarely observed in the literature, but it is clinically relevant. Overlapping symptoms can obscure localization and delay diagnosis, making electrodiagnostic testing essential for differentiation. A previously reported case involved a 55-year-old female with a history of hepatitis C and IV drug use, who was found to have both CTS and GCS after presenting to the emergency department with a two-day history of redness, swelling, pain, and numbness in all five digits of her right hand. After imaging, laboratory work, and eventual surgery, she was found to have pseudogout, entrapping both the median nerve at the carpal tunnel and the ulnar nerve at Guyon’s canal. This study was one of the earliest reports of both median and ulnar nerve compression at their respective wrist entrapment points [5]. It shows how distinct pathologies can co-occur and complicate clinical assessment.

Electrodiagnostic testing, including specific testing of the dorsal ulnar cutaneous nerve (DUCN), can help differentiate if the ulnar nerve entrapment site is more distal or proximal. In one report, a patient with double crush syndrome entrapping the ulnar nerve at both the cubital tunnel and Guyon’s canal presented with right-hand weakness, dorsal forearm numbness, and numbness in the fourth and fifth digits. This was assumed to be cubital tunnel syndrome and led to the surgical release of the ulnar nerve. While this surgery improved the numbness in the dorsal forearm, the hand weakness and numbness persisted. This was because the DUCN was freed of its entrapment proximally, but the nerve was still compressed at Guyon’s canal, causing the hand symptoms. A second surgery was then performed to release the ulnar nerve entrapment at Guyon’s canal to relieve the remaining symptoms [6]. The DUCN is one of the terminal branches off the ulnar nerve before Guyon’s canal, which means it is commonly used as an ulnar nerve reference point to differentiate entrapment at Guyon’s canal from a more proximal entrapment [6]. While CTS and GCS are common individually, their simultaneous occurrence is rare, making clinical localization challenging.

In this article, we report a patient with concurrent ulnar and median nerve entrapment at the wrist, without a single compressive local lesion to explain both findings. The patient’s history, including prior cervical spine surgery, was reviewed and taken into consideration during the diagnostic process. We highlight the value of electrodiagnostic testing and DUCN testing in confirming dual entrapment and guiding management. We also validate our findings against standardized clinical practices and compare them to other similar findings in the literature.

## Case presentation

A 54-year-old female presented with the chief complaint of numbness and tingling in both hands, worse on the right. She has a history of a cholecystectomy, asthma, narcolepsy, sinus issues, left Morton’s neuroma excision, right wrist ganglion incision, left deQuervain’s surgery, C5-7 ACDF, and C6 corpectomy four months before the electrodiagnostic study. A postoperative cervical spine X-ray showed C5-C7 fusion with mild spondylolisthesis on C4-C5, as well as correct hardware placement. She denied any history of diabetes, tobacco use, or chemotherapy. Her symptoms had worsened over the past year and were exacerbated by typing, driving, and sleeping. She had not been wearing a wrist splint at night but found relief with rest and hydrocodone/acetaminophen 10/325 every six hours as needed. Her history and prior cervical spine imaging were reviewed to rule out proximal causes of neuropathy.

On physical exam, there was no visible hand atrophy. She had a full range of motion in her neck, negative Spurling’s bilaterally, negative Hoffmann’s bilaterally, and negative Tinel’s bilaterally at the wrists and elbows. Her neurological examination revealed intact sensation throughout the upper extremities, full strength in all muscle groups bilaterally, and 1+/4 deep tendon reflexes at the biceps, brachioradialis, and triceps bilaterally.

In the electrodiagnostic study, nerve conduction testing revealed that the right median sensory latency was normal compared to the reference value. However, latency was prolonged by 0.5 ms at the right median sensory nerve at the wrist with pickup at the second digit compared to the right ulnar sensory nerve with pickup at the fifth digit (Table 1). Right ulnar sensory amplitude was diminished across the wrist, but latency was normal (Table 1). The DUCN was found to have a normal latency and amplitude. The left mixed median sensory nerve and the left mixed ulnar sensory nerve were compared across the wrist with pickup at the fourth digit. This study did not show a comparative prolonged latency of at least 0.4 ms (Table 1). Left median sensory, bilateral median motor, left ulnar sensory, bilateral ulnar motor, and right radial sensory were all found to have normal latencies, amplitudes, and conduction velocities (Table 2).

**Table 1:** Sensory nerve conduction study results

Nerve/sites	Recording site	Peak latency (ms)	Amplitude (μV)	Segments	Distance (cm)
Right median - digit II (antidromic)					
Wrist	Digit II	3.6	27	Wrist - digit II	14
Reference	X	≤3.6	≥15	Reference	X
Left median - digit II (antidromic)					
Wrist	Digit II	3.4	57.8	Wrist - digit II	14
Reference	X	≤3.6	≥15	Reference	X
Right ulnar - digit V (antidromic)					
Wrist	Digit V	3.1	11.1	Wrist - digit V	14
Reference	X	≤3.7	≥15	Reference	X
Left ulnar - digit V (antidromic)					
Wrist	Digit V	3.2	49.3	Wrist - digit V	14
Reference	X	≤3.7	≥15	Reference	X
Right radial - thumb					
Forearm	Thumb	2.2	38.8	Forearm - thumb	10
Reference	X	≤2.9	≥15	Reference	X
Left median, ulnar - ring finger comparison					
Median wrist	Ring finger	3.4	19.7	Median wrist - ring finger	14
Ulnar wrist	Ring finger	3.4	17.7	Ulnar wrist - ring finger	14
Right dorsal ulnar cutaneous - hand dorsum (forearm)					
Forearm	Hand dorsum	2.5	8.5	Forearm - hand dorsum	10
Reference	X	≤2.9	≥5.0	Reference	X

**Table 2:** Motor nerve conduction study results APB: abductor pollicis brevis, ADM: abductor digiti minimi

Nerve/sites	Muscle	Latency (ms)	Amplitude (μV)	Amp %	Segments	Distance (cm)	Velocity (m/s)
Right median - APB							
Wrist	APB	3.7	9.5	100	Wrist - APB	8	X
Reference	X	≤4.2	≥5.0	X	Reference	X	X
Left median - APB							
Wrist	APB	2.8	9	100	Wrist - APB	8	X
Reference	X	≤4.2	≥5.0	X	Reference	X	X
Right ulnar - ADM							
Wrist	ADM	2.9	5.7	100	Wrist - ADM	8	X
Reference	X	≤4.2	≥3.0	X	Reference	X	X
Below elbow	ADM	5	6.8	119	Below elbow - wrist	23	109
Reference	X	X	X	X	Reference	X	≥50
Above elbow	ADM	7.3	5.9	103	Above elbow - below elbow	13	57
Reference	X	X	X	X	Reference	X	≥50
Left ulnar - ADM							
Wrist	ADM	2.8	6.5	100	Wrist - ADM	8	X
Reference	X	≤4.2	≥3.0	X	Reference	X	X
Below elbow	ADM	5.5	6.3	97.5	Below elbow - wrist	24	88
Reference	X	X	X	X	Reference	X	≥50
Above elbow	ADM	7.1	5.5	85.6	Above elbow - below elbow	11	70
Reference	X	X	X	X	Reference	X	≥50

Needle electromyography revealed polyphasic motor unit potentials within the right deltoid (Table 3). No spontaneous activity, amplitude abnormalities, polyphasic potentials, or recruitment abnormalities were found in the left deltoid, bilateral biceps, bilateral triceps, bilateral pronator teres, bilateral first dorsal interossei, or right abductor pollicis brevis.

**Table 3:** Electromyography test results IA: insertion activity, PSW: positive sharp wave, MUAP: motor unit action potential, PPP: polyphasic potentials

X	Spontaneous					MUAP			Recruitment
Muscle	IA	Fibrillations	PSW	Fasciculations	Other	Amplitude	Duration	PPP	Pattern
Left deltoid	Normal	None	None	None	None	Normal	Normal	None	Normal
Left biceps brachii	Normal	None	None	None	None	Normal	Normal	None	Normal
Left triceps brachii	Normal	None	None	None	None	Normal	Normal	None	Normal
Left pronator teres	Normal	None	None	None	None	Normal	Normal	None	Normal
Left first dorsal interosseous	Normal	None	None	None	None	Normal	Normal	None	Normal
Right deltoid	Normal	None	None	None	None	Normal	Normal	1+	Normal
Right biceps brachii	Normal	None	None	None	None	Normal	Normal	None	Normal
Right triceps brachii	Normal	None	None	None	None	Normal	Normal	None	Normal
Right pronator teres	Normal	None	None	None	None	Normal	Normal	None	Normal
Right first dorsal interosseous	Normal	None	None	None	None	Normal	Normal	None	Normal
Right abductor pollicis brevis	Normal	None	None	None	None	Normal	Normal	None	Normal

This patient was diagnosed with mild right-sided CTS, mild right-sided ulnar nerve entrapment at Guyon’s canal, and chronic right-sided cervical radiculopathy at C5. Her discharge plan of care consisted of continuing to wear a wrist splint at night and following up with an orthopedic hand surgeon for management of her symptoms.

## Discussion

This patient had a rare case of simultaneous GCS and CTS in the setting of a previous cervical spine surgery. Electrodiagnostic testing, including DUCN evaluation, was crucial in confirming the diagnosis and localization of ulnar nerve involvement, which can often be overlooked. The right median sensory latency was prolonged by 0.5 ms compared to the ulnar sensory nerve, consistent with mild CTS. The right ulnar sensory amplitude was reduced across the wrist, supporting ulnar nerve entrapment at Guyon’s canal. However, the patient's main complaints were numbness and tingling in the right hand, which was greater than in the left. Electrodiagnostic testing localized sites of entrapment at the wrist for both the median and ulnar nerves, aligning with her symptom distribution. The etiology of this patient’s symptoms would have been extremely difficult to identify without electrodiagnostic testing, given the low prevalence of ulnar nerve entrapment at Guyon’s canal. Ulnar nerve entrapment at Guyon’s canal has been reported in a few cases to be secondary to various pathologies such as cysts, pseudogout, or repetitive trauma [4]. The absence of a mass lesion or trauma distinguishes this patient's presentation from prior reports. One case hypothesized that ulnar nerve entrapment was a sequela of recurrent CTS [7]. In this patient, recurrent CTS, cervical radiculopathy, prior cervical spine surgery, and systemic causes like diabetes or hypothyroidism were evaluated and excluded as the primary etiology, given the patient’s normal physical exam, lack of systemic disease, and localized electrodiagnostic findings.

Since combined CTS and GCS is rare, the DUCN was assessed to ensure proper interpretation of results. The DUCN, one of the final branches of the ulnar nerve before Guyon’s canal, is commonly used to differentiate between ulnar nerve entrapment at Guyon’s canal versus the cubital tunnel [8]. In our study, the DUCN exhibited normal amplitude and latency, indicating that the lesion was distal to this branch, confirming localization to Guyon's canal.

Anatomical variants at Guyon’s canal can be a cause of ulnar entrapment without an obvious pathology. Since there were no historical findings to suggest typical pathology, variant pathology should be considered. Studies have shown that between 20-35% of people were found to have an aberrant muscle crossing Guyon’s canal, giving it the title of an accessory abductor digiti minimi [9,10]. This variant muscle is more common in males and is found bilaterally in 50% of cases; however, it is often asymptomatic [11].

In this case, preservation of the DUCN function supported distal ulnar nerve compression, which indicated GCS. Electrodiagnostic testing played a crucial role in differentiating overlapping neuropathies and excluding proximal causes. This case highlights the diagnostic value of comprehensive testing in patients presenting with neuropathic symptoms, especially when prior surgical or structural etiologies are part of the differential.

## Conclusions

This case highlights a rare clinical presentation of concurrent CTS and GCS without an identifiable local lesion. It also highlights the diagnostic importance of comprehensive electrodiagnostic testing, including evaluation of the DUCN, in identifying concurrent entrapment neuropathies that may be overlooked clinically. Additionally, the evaluation of the DUCN is a clinically valuable tool for determining the location of pathology. These practices, when combined, could facilitate the making of accurate diagnoses to guide patient care. Providers should be aware of the importance of comprehensive diagnostic workups when symptoms overlap between multiple neuropathies. Even in the absence of classic risk factors or imaging abnormalities, clinicians should be aware of unusual presentations. Further studies of anatomical variants and other risk factors may help identify patients at risk for concurrent entrapment neuropathies and guide their management strategies.
